# Biomechanical evaluation of combined short segment fixation and augmentation of incomplete osteoporotic burst fractures

**DOI:** 10.1186/1471-2474-14-360

**Published:** 2013-12-21

**Authors:** René Hartensuer, Dominic Gehweiler, Martin Schulze, Lars Matuszewski, Michael J Raschke, Thomas Vordemvenne

**Affiliations:** 1 Department of Trauma-, Hand-, and Reconstructive Surgery, Westfälische Wilhelms-University Münster, Albert-Schweitzer-Campus 1, W1, Münster 48149, Germany; 2 Department of Clinical Radiology, Westfälische Wilhelms-University Münster, Albert-Schweitzer-Campus 1, A1, Münster 48149, Germany

**Keywords:** Biomechanics, Osteoporosis, Trauma, Vertebroplasty, Instrumentation, Burst fracture, Spine

## Abstract

**Background:**

Treating traumatic fractures in osteoporosis is challenging. Multiple clinical treatment options are found in literature. Augmentation techniques are promising to reduce treatment-related morbidity. In recent years, there have been an increasing number of reports about extended indication for augmentation techniques. However, biomechanical evaluations of these techniques are limited.

**Methods:**

Nine thoracolumbar osteoporotic spinal samples (4 FSU) were harvested from postmortem donors and immediately frozen. Biomechanical testing was performed by a robotic-based spine tester. Standardized incomplete burst fractures were created by a combination of osteotomy-like weakening and high velocity compression using a hydraulic material testing apparatus. Biomechanical measurements were performed on specimens in the following conditions: 1) intact, 2) fractured, 3) bisegmental instrumented, 4) bisegmental instrumented with vertebroplasty (hybrid augmentation, HA) and 5) stand-alone vertebroplasty (VP). The range of motion (RoM), neutral zone (NZ), elastic zone (EZ) and stiffness parameters were determined. Statistical evaluation was performed using Wilcoxon signed-rank test for paired samples (p = 0.05).

**Results:**

Significant increases in RoM and in the NZ and EZ (p < 0.005) were observed after fracture production. The RoM was decreased significantly by applying the dorsal bisegmental instrumentation to the fractured specimens (p < 0.005). VP reduced fractured RoM in flexion but was still increased significantly (p < 0.05) above intact kinematic values. NZ stiffness (p < 0.05) and EZ stiffness (p < 0.01) was increased by VP but remained lower than prefracture values. The combination of short segment instrumentation and vertebroplasty (HA) showed no significant changes in RoM and stiffness in NZ in comparison to the instrumented group, except for significant increase of EZ stiffness in flexion (p < 0.05).

**Conclusions:**

Stand-alone vertebroplasty (VP) showed some degree of support of the anterior column but was accompanied by persistent traumatic instability. Therefore, we would advocate against using VP as a stand-alone procedure in traumatic fractures.

HA did not increase primary stability of short segment instrumentation. Some additional support of anterior column and changes of kinematic values of the EZ may lead one to suppose that additive augmentation may reduce the load of dorsal implants and possibly reduce the risk of implant failure.

## Background

Incomplete burst fractures in osteoporotic patients are one of the most upcoming and challenging issues in spinal traumatology and optimal treatment remains an unresolved question. So far there is a paucity of randomized control trials as well as biomechanical studies which address the question of appropriate treatment and the understanding of the mechanical stability of these common injuries. In patients with good bone quality, recommended treatment options vary from conservative treatment [1] to combined posterior-anterior fusion [2]. In osteopenic or even osteoporotic patients with traumatic incomplete burst fractures, treatment options are even more manifold, but again without sufficient supporting evidence for positive outcomes.

In case of neurologically and posterior ligament intact fractures with low-grade destruction of the vertebral body, a panel of leading spine surgeons (The Spine Trauma Study Group) recommended a posterior approach [3].

In elderly patients, fractures are frequently accompanied by comorbidities, so that the anterior thoracic, retroperitoneal or abdominal approach is not preferable. Nevertheless, particularly in this patient population, prolonged immobilization needs to be averted and immediate primary stability is aspired [4].

Several factors, including the physiological stress to the patient, morbidity and complication rates need to be taken into account in the choice of surgical approach [5]. Even if an anterior reconstruction could have been considered, a single posterior approach is recommended in the elderly with poor bone quality [4].

However, it is known that conventional pedicle screws or other posterior stabilization strategies may fail more often in osteoporotic patients because of the poor bone-implant interface [6]. This effect may even be worse if there is a lack of support for the anterior column [7]. Aside from the already discussed mortality in older patients from anterior surgery, conventional anterior constructions with cages may also fail. Relatively hard cages from titanium, polyether-ether ketone (PEEK) or other materials may easily breach into the endplate. This may lead to loss of correction, increase of bearing load to the posterior implants or even in complete implant failure [8].

Conscious of these mechanical problems in osteoporotic bone and the lack of evidence supporting any treatment, the most recent publications describe a stand-alone cement augmentation in different techniques such as vertebroplasty or kyphoplasty and augmentation support of conventional instrumentation with a dorsal approach only [9].

Kyphoplasty is considered to be a relatively safe and effective treatment of osteoporotic thoracolumbar burst fractures. It is reported to be able to treat pain, restore vertebral body height, and reduce kyphosis without deterioration of fragment retropulsion [10]. Also, vertebroplasty (VP) as a stand-alone procedure is reported to be safe in burst fractures [11]. Like kyphoplasty, VP reportedly is able to restore vertebral body height and to reasonably correct kyphotic deformity. In addition, there are also biomechanical indications that cement injection might restore mechanical properties of the fractured vertebra [12]. Therefore, some authors appraise VP as being adequate in eliminating the need for major operations [13]. However, to our knowledge, further biomechanical investigation on kinematic effects of cement augmentation in incomplete burst fractures in osteoporosis is lacking. Also, the clinical value of the available reports is questionable in terms of evidence-based treatment.

Several published clinical reports describe a form of hybrid technique (hybrid augmentation, HA) by combining a dorsal short segment pedicle screw construction with an augmentation of the fractured vertebra [7,14-20]. By augmenting the fractured vertebra, a stabilization of the anterior column is anticipated.

The HA technique seems to combine the advantages of 2 relatively less invasive procedures to treat this kind of fractures even in older and potentially osteoporotic patients where isolated dorsal instrumentation would possibly fail. The technique is expected to reduce kyphotic loss and instrumentation failure in comparison to stand-alone dorsal instrumentation [21]. It is also expected to decrease the inherent morbidity, blood loss, operative time, length of the hospital stay, and costs associated with an anterior thoracic or abdominal approach [18]. Complete percutaneous treatment using HA has also been reported in literature [16] but there is little knowledge on the biomechanical effects of this technique [7].

Having been premised on the lack of conceptual clarity of treatment in incomplete burst fractures in osteoporosis and based on the little biomechanical knowledge we have so far, this study was designed to gain kinematic awareness in this challenging question using a validated method that experimentally inflicts incomplete burst fractures [22].

The objective of this study is to investigate the kinematic effects of different dorsal augmentation-related treatment options of incomplete burst fractures in osteoporosis.

## Methods

### Specimens

Nine spinal samples consisting of 5 vertebrae (4 functional spinal units, FSU) were harvested from postmortem donors in our anatomical institute and immediately frozen. All specimens were taken from the thoracolumbar junction. The median age of the specimens was 75 (Q1: 73; Q3: 87.5) years, with nearly equal sex distribution (male: female = 4:5). The ethics commission for the medical chamber of Westfalen-Lippe and the medical faculty of the University of Muenster approved the usage of post mortem samples of the local anatomical institution.

In all samples, bone mineral density (BMD) was measured using quantitative computed tomography (Q-CT) [23]. The median BMD was 103 (Q1: 70.9; Q3: 117.7) mg Ca-HA/mL and the median T-score of -2.7 (Q1: -3.57; Q3: -1.84) was calculated. Except for one sample, only osteoporotic or osteopenic spine samples were available (Table 1). Just before testing, all specimens were thawed slowly to room temperature. All soft tissue and muscles were dissected carefully to preserve the osseous and ligamentous structures.

**Table 1:** Overview of specimens and their important characteristics

**Test number**	**Age**	**Fracture level**	**Fracture load (kN)**	**Sex**	**BMD (mg Ca-HA/ml)**	**T-score**
1	80	Th 12	3,9	Male	62.2	-4.3
2	88	Th 11	5,4	Female	143	-0.6
3	73	L 1	3,5	Male	112.7	-2.3
4	75	L 1	2,7	Male	111.1	-2.4
5	73	L 1	3,3	Male	103	-2.7
6	89	L 1	5,3	Female	40.7	-4.3
7	87	L 1	2,9	Female	79.5	-2.9
8	75	L 1	1,9	Female	84.7	-2.7
9	71	Th 12	4,8	Female	122.7	-1.3

The caudal and cranial vertebral body was rigidly fixed in a standardized manner into a piece of plastic pipe filled with 2-component resin embedding system (Technovit® 3040 Kulzer Heraeus GmbH, Germany). This setup could then be attached to customized tools to mount the samples into the servo hydraulic testing machine and the testing robot.

All samples were kept moist during the dissection and testing process.

### Fracture creation

The presented procedure took advantage of the published classical approaches to study burst fractures that utilized spine fragments mounted onto a fracture apparatus [24-28]. All known methods were not able to specifically produce incomplete burst fractures, and thus needed to be refined for this study.

According to a validated testing setup, a standardized osteotomy was performed and temporarily transfixed. All specimens were mounted into a hydraulic material testing apparatus (Instron 8874, Instron Structural Testing Systems GmbH, Germany) in a 10° flexion angle. The specimens were then axially compressed under displacement control with a speed of 300 mm/s until the vertical distance was reduced to 20% of the original target vertebral body height. Detailed presentation of this technique is published elsewhere [22].

### Instrumentation and augmentation

The posterior instrumentation was performed bisegmentally using the USS Fracture System (Synthes GmbH, Switzerland). USS Schanz screws with 6.2 mm dual core were used. No additional force by reduction due to fracture clamp was applied in consideration of the bone quality of the samples used.

**Figure 1:**
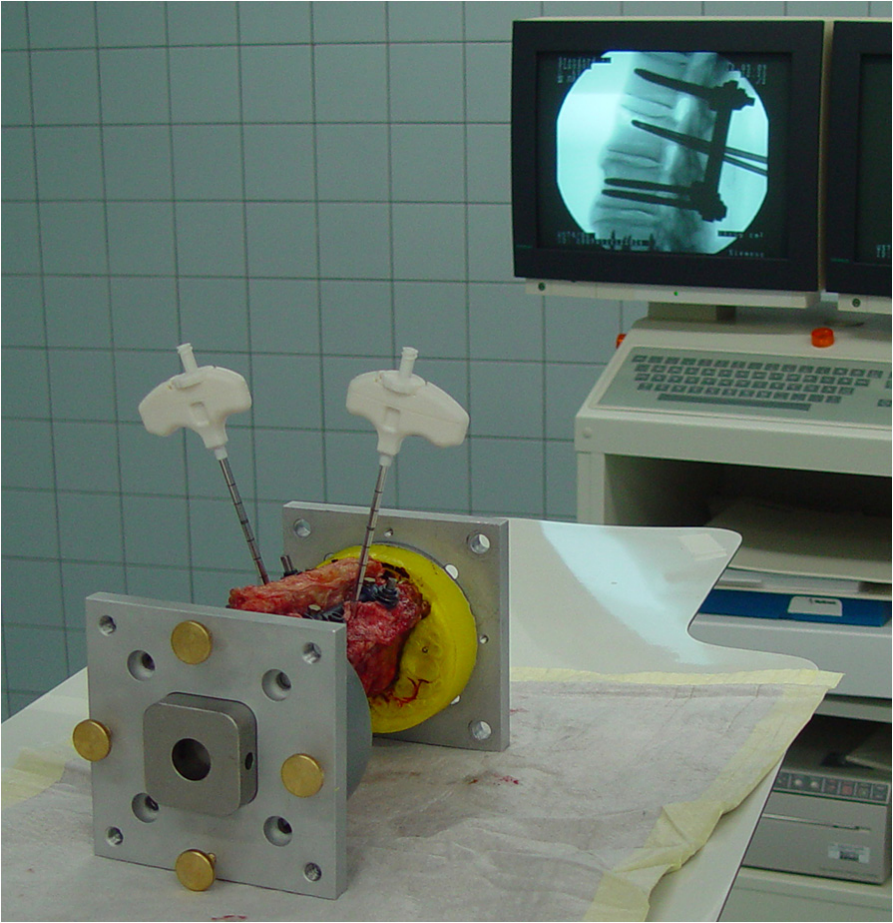
**Experimental setup of in vitro vertebroplasty (VP) for hybrid augmentation (HA).** After fracture creation, human thoracolumbar postmortem specimens were bisegmentally instrumented and additionally cement-augmented under fluoroscopic control.

**Figure 2:**
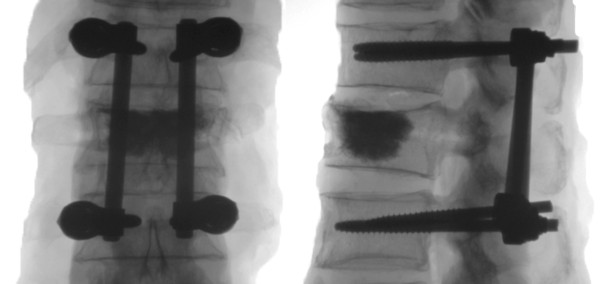
**Fluoroscopic control after HA.** The posterior instrumentation was performed bisegmentally and by biportal transpedicular VP.

VP was realized using PMMA (Vertecem, Synthes GmbH, Switzerland) according to the recommended surgical biportal transpedicular technique under fluoroscopic control (Figures 1 and 2). Cement was mixed according to the manufacturer’s recommendations. All procedures were performed at room temperature. Before kinematic testing, the complete hardening of the cement at room temperature was ensured. The median volume of the injected cement was 5.7 mL (Q1: 4.3 mL; Q3: 8.2 mL).

### Kinematic testing

The spine testing facility used in this study was based on a 6° of freedom robotic arm (KUKA/KR125, Kuka Augsburg, Germany) which enabled execution of complex motion patterns. The robot was used to manipulate the specimen via predefined load (7.5 Nm, pure moments) on the cranial vertebrae. A sensitive force/torque sensor (Theta SI-1500-240 from ATI Industrial Automation, USA) was mounted at the robot’s end-effector which enabled simultaneous measurements of applied forces and torques during load-controlled robot movement according to a standardized and evaluated robotic-based setup [29].

All spine samples were subjected to moments in flexion-extension, lateral bending and axial rotation in the following 5 different states:

1) intact specimen

2) after fracture induction

3) after bisegmental instrumentation

4) HA: combined bisegmental instrumentation and VP

5) stand-alone VP

For each group of specimens, the range of motion (RoM), neutral zone (NZ) and elastic zone (EZ), and the stiffness of the NZ and EZ were determined. In extension–flexion, the combined motion was evaluated for extension and flexion separately. Therefore, zero-crossing was defined to be half of NZ.

### Statistics

Statistical analysis was performed using the Wilcoxon signed-rank test for paired samples at the significance level of p = 0.05. This nonparametric test is suitable for analyzing data that may not be normally distributed. In addition, a repeated measures analysis was performed.

The calculation of the statistics was performed with software programmed in C# (Visual C#, Microsoft Corporation, USA). This software is based on ALGLIB® (ALGLIB project, Russian Federation) and was validated using SPSS® (SPSS® Statistics, IBM®, USA).

Sample size was limited by the availability of human postmortem samples but was comparable to sample sizes used in similar studies [7,30] and cited in published recommendations [31].

## Results

In all samples, a fracture resulted in the target vertebral body by performing a single compression cycle. Yield strengths recorded during the fracture tests are shown in Table 1 and ranged from 1.9 kN to 5.4 kN.

**Figure 3:**
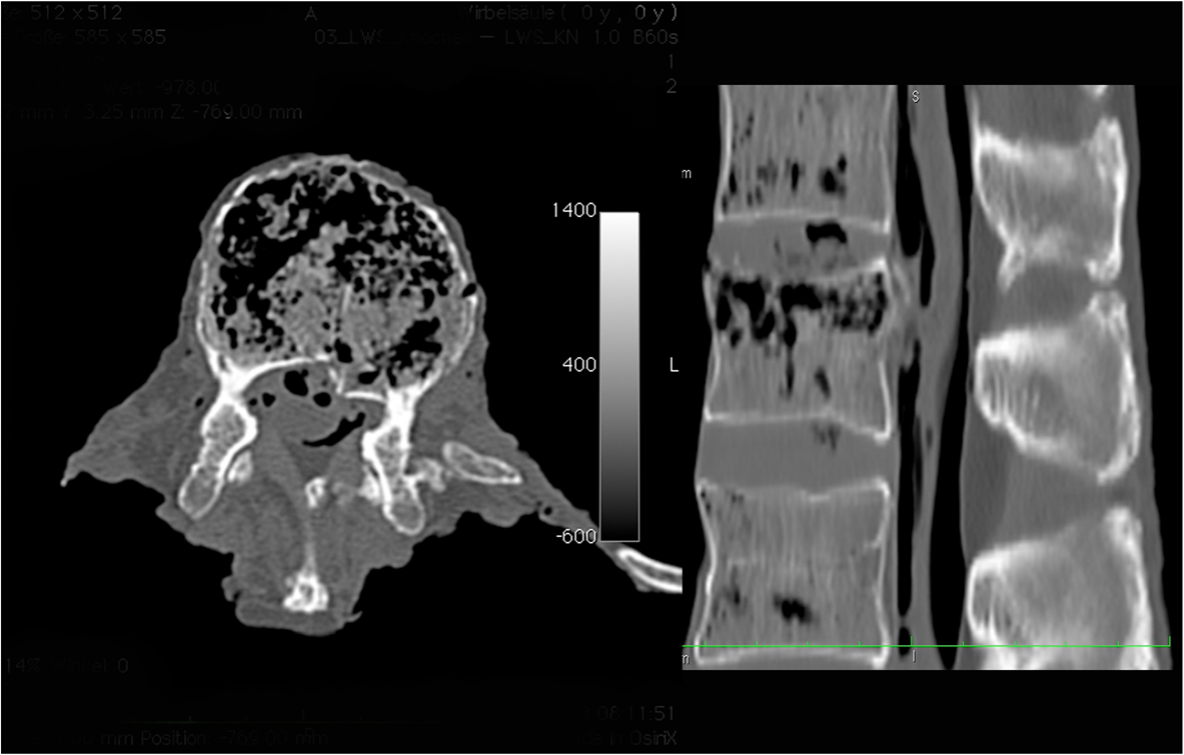
**CT scan after fracture infliction, axial and sagittal plane.** Incomplete burst fracture with involvement of anterior and middle column was produced in all used specimens.

Evaluations via CT scan and macroscopic inspection of the specimens showed no signs of injury to the facets or posterior ligamentous complex (PLC) or rotational injury. An independent senior spine surgeon and a senior radiologist identified a superior incomplete burst fractures in all samples (Figure 3).

All specimens presented a significant increase of the measured RoM (p < 0.005) for all directions by induction of the cranial burst fracture in comparison to intact kinematic behavior. The RoM was decreased significantly (p < 0.005) by applying the dorsal bisegmental instrumentation to levels similar to the intact values. The NZ presented effects similar to those observed for RoM. After fracture infliction, the NZ increased significantly (p < 0.01).

An adequate screw positioning after posterior instrumentation was checked and documented via fluoroscopy.

After dorsal bisegmental instrumentation, the NZ was again reduced significantly (p < 0.005). No statistically significant differences were seen when comparing the NZ of intact 4 segmental specimens and bisegmentally instrumented fractured 4 segmental specimens.

VP was successfully performed in all specimens and an adequate cement distribution was observed by fluoroscopy.

### Kinematic effects on primary stability by stand-alone vertebroplasty (VP)

#### Flexion

**Figure 4:**
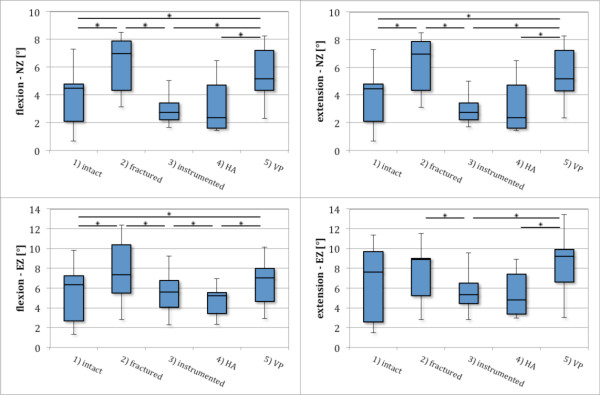
**Comparison of RoM values for five specimen groups in extension and flexion in neutral zone (NZ) and elastic zone (EZ)*.*** Incomplete burst fracture creation (2) resulted in a significant increase in NZ and EZ, posterior bisegmental instrumentation resulted in a significant decrease of NZ and EZ (3) for flexion and extension. HA resulted in no additional primary stability (4), whereas isolated PMMA VP (5) increased fractured values, but remained significant less than prefractured values in flexion (NZ and EZ) as well as NZ in extension.

In flexion, fracture significantly increased NZ of the intact specimen by 57% (p < 0.005). EZ was also increased significantly (p < 0.01) after fracture by 16%. VP reduced this effect for NZ but was still increased significantly by 16% of intact values (p < 0.05). EZ was also reduced by VP but remained 11% significantly (p < 0.05) above intact kinematic values (Figure 4, Table 2).

**Table 2:** Biomechanical values (median, (Q1, Q3)) in flexion and extension in all groups

**Flexion**	**NZ**		**EZ**		**sNZ**		**sEZ**	
	**[°]**		**[°]**		**[°]**		**[°]**	
1) Intact specimen	4.5 (2.1, 4.8)	*100%*	6.4 (2.7, 7.3)	*100%*	0.32 (0.20, 0.63)	*100%*	1.17 (0.89, 1.67)	*100%*
2) Fractured	7.0 (4.3, 7.9)	*157%*	7.4 (5.5, 10.4)	*116%*	0.14 (0.12, 0.15)	*44%*	1.01 (0.84, 1.15)	*86%*
3) Biseg. instrument.	2.7 (2.2, 3.4)	*61%*	5.6 (4.1, 6.7)	*88%*	0.53 (0.34, 0.60)	*165%*	0.93 (0.90, 1.13)	*80%*
4) HA	2.3 (1.6, 4.7)	*53%*	5.2 (3.4, 5.5)	*82%*	0.60 (0.32, 0.64)	*187%*	1.18 (1.08, 1.48)	*101%*
5) VP	5.2 (4.3, 7.2)	*116%*	7.0 (4.6, 8.0)	*111%*	0.16 (0.14, 0.28)	*51%*	1.10 (0.99, 1.44)	*94%*
**Extension**	**NZ**		**EZ**		**sNZ**		**sEZ**	
	**[°]**		**[°]**		**[°]**		**[°]**	
1) Intact specimen	4.5 (2.1, 4.8)	*100%*	7.6 (2.5, 9.7)	*100%*	0.29 (0.22, 0.62)	*100%*	0.89 (0.79, 2.04)	*100%*
2) Fractured	7.0 (4.3, 7.9)	*157%*	8.9 (5.2, 9.0)	*117%*	0.15 (0.12, 0.19)	*54%*	0.95 (0.84, 1.06)	*107%*
3) Biseg. instrument.	2.7 (2.2, 3.4)	*61%*	5.3 (4.5, 6.5)	*70%*	0.44 (0.30, 0.62)	*153%*	1.12 (0.94, 1.23)	*126%*
4) HA	2.3 (1.6, 4.7)	*53%*	4.8 (3.4, 7.4)	*63%*	0.49 (0.34, 0.57)	*170%*	1.23 (1.06, 1.44)	*138%*
5) VP	5.2 (4.3, 7.2)	*116%*	9.2 (6.6, 9.9)	*121%*	0.16 (0.14, 0.19)	*55%*	0.91 (0.88, 1.10)	*102%*

In addition, all data are also presented normalized by the intact performance (as a percentage of prefractured values).

**Figure 5:**
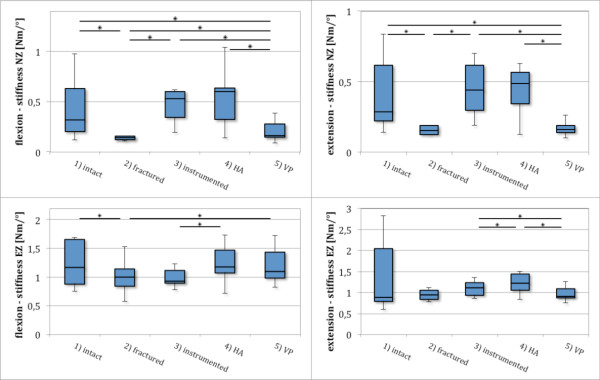
**Comparison of stiffness values for five specimen groups in extension and flexion in NZ (sNZ) and EZ (sEZ).** Incomplete burst fracture creation (2) resulted in a significant decrease of sNZ and sEZ in flexion as well as sNZ in extension. Posterior bisegmental instrumentation (3) restored sNZ but was not able to restore prefractured sEZ values in flexion. Hybrid augmentation (HA) takes advantage of changes in stiffness parameters by instrumentation (4). In addition, a significant change compared to fractured values of sEZ even for flexion was observed following HA. Stand-alone VP (5) increased sNZ on flexion and remained significantly lower than pre-fracture values. VP significantly increased sEZ in flexion compared to the fractured (2) state to reach approximately pre-fractured values.

Fracture also significantly decreased NZ stiffness (p < 0.005) to 44% and EZ stiffness to 86% of the intact specimen (p < 0.005). VP significantly increased NZ stiffness (p < 0.05) to 51% and EZ stiffness (p < 0.01) to 94%, but stiffness parameters remained lower than prefracture values (Figure 5, Table 2).

#### Extension

In extension, values for NZ were identical with flexion, caused by the defined zero crossing. EZ was increased by the fracture by 17% and this increase was not clearly influenced by VP. Stiffness in neutral zone (sNZ) was significantly reduced by the fracture (p < 0.05), whereas no influence of stiffness in elastic zone (sEZ) was seen after fracture creation. No perceptible change was seen from VP for sNZ and sEZ compared to the fractured state (Figures 4 and 5, Table 2).

#### Lateral bending

In lateral bending, NZ was increased significantly (p < 0.005) by more than twice the intact kinematics after fracture creation. Also EZ was increased significantly (p < 0.005) by 28%. VP reduced NZ increase but remained 79% significantly (p < 0.005) more than the intact values. The increase of EZ could not be influenced by VP.

**Figure 6:**
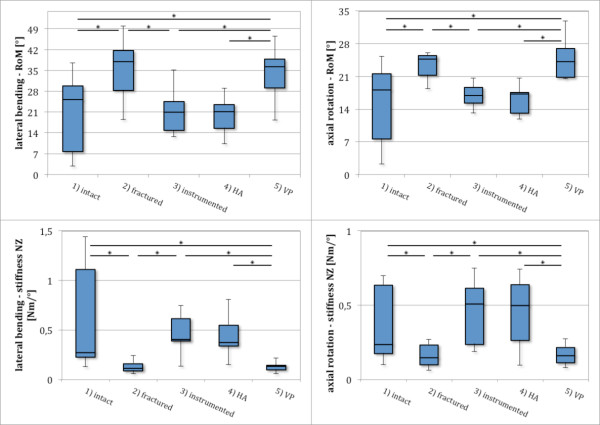
**Comparison of kinematic values for five specimen groups in lateral bending and axial rotation.** Incomplete burst fracture creation (2) resulted in a significant increase in RoM and decrease of stiffness in neutral zone (sNZ) and elastic zone (sEZ). Posterior bisegmental instrumentation (3) restored prefractured kinematic values in lateral bending and axial rotation. Hybrid augmentation (HA) resulted in no additional increase in stiffness or further reduction of RoM (4). Stand-alone VP (5) did not alter fractured values for lateral bending and axial rotation.

Stiffness in NZ and EZ remained significantly reduced by approximately half the prefractured values after VP for NZ stiffness (p < 0.01) and three quarters for EZ stiffness (p < 0.05) (Figure 6, Table 3).

**Table 3:** Biomechanical values (median, (Q1, Q3)) for lateral bending and axial rotation in all groups

**Axial rotation**	**NZ**		**EZ**		**sNZ**		**sEZ**	
	**[°]**		**[°]**		**[°]**		**[°]**	
1) Intact specimen	4.2 (2.2, 5.9)	*100%*	13.0 (5.8, 15.8)	*100%*	0.24 (0.18, 0.64)	*100%*	1.10 (1.06, 2.52)	*100%*
2) Fractured	6.8 (6.4, 8.0)	*161%*	16.7 (15.3, 18.4)	*129%*	0.15 (0.10, 0.23)	*71%*	0.93 (0.83, 1.12)	*85%*
3) Biseg. instrument.	4.1 (3.2, 5.2)	*97%*	13.2 (12.1, 14.3)	*102%*	0.51 (0.24, 0.61)	*214%*	0.99 (0.87, 1.13)	*90%*
4) HA	3.4 (3.0, 4.9)	*81%*	12.5 (8.9, 14.6)	*96%*	0.50 (0.26, 0.64)	*209%*	1.10 (0.94, 1.26)	*101%*
5) VP	6.6 (5.5, 7.5)	*156%*	16.6 (15.0, 19.7)	*128%*	0.16 (0.11, 0.22)	*67%*	0.95 (0.84, 1.08)	*87%*
**Lateral bending**	**NZ**		**EZ**		**sNZ**		**sEZ**	
	**[°]**		**[°]**		**[°]**		**[°]**	
1) Intact specimen	9.3 (3.9, 14.6)	*100%*	12.8 (3.8, 17.0)	*100%*	0.28 (0.23, 1.11)	*100%*	0.73 (0.62, 1.40)	*100%*
2) Fractured	19.8 (13.1, 20.5)	*213%*	16.4 (12.0, 19.2)	*128%*	0.12 (0.09, 0.16)	*42%*	0.62 (0.51, 0.74)	*85%*
3) Biseg. instrument.	8.2 (6.4, 12.7)	*88%*	12.1 (8.5, 14.4)	*94%*	0.41 (0.39, 0.62)	*147%*	0.75 (0.63, 0.90)	*103%*
4) HA	8.0 (6.2, 13.1)	*86%*	11.9 (8.0, 14.1)	*93%*	0.38 (0.34, 0.55)	*136%*	0.73 (0.65, 0.90)	*99%*
5) VP	16.6 (13.5, 20.6)	*179%*	17.1 (13.7, 21.0)	*134%*	0.14 (0.10, 0.15)	*50%*	0.63 (0.57, 0.81)	*86%*

In addition, all data are also presented normalized by the intact performance (as a percentage of prefractured values).

#### Axial rotation

In axial rotation, NZ was increased significantly by 61% (p < 0.01), and EZ by 29% (p < 0.005) after fracture creation. There was no distinct change of this increase in NZ and EZ caused by VP. NZ stiffness was reduced significantly (p < 0.01) by 29%, and EZ stiffness significantly (p < 0.005) by 15%. After VP, values persisted approximately at the fracture value level (Figure 6, Table 3).

### Kinematic effects on primary stability by hybrid augmentation (HA)

#### Flexion

Instrumentation reduced intact NZ to 61% in flexion. This effect was incremented by HA to 53% of intact values. Also, EZ was influenced by instrumentation to 88% of intact kinematics and to 82% by HA. The additional influence in HA was not significant (Figure 4, Table 2).

Instrumentation and HA also increased NZ stiffness above intact levels. No significant influence was seen after HA in comparison to instrumentation. In EZ, instrumentation was not able to restore intact stiffness. Here, a significant (p < 0.05) increase in sEZ was observed in HA compared to instrumentation. Intact stiffness values were approximately restored by a combination of instrumentation and cement augmentation (Figure 5, Table 2).

#### Extension

In extension, instrumentation and HA reduced motion in NZ and EZ below the prefracture values. Also, stiffness parameters in NZ and EZ were increased above intact kinematics. No significant changes between instrumentation and HA were observed in extension (Figures 4 and 5, Table 2).

#### Lateral bending

Movement in NZ and EZ was reduced by instrumentation and HA below the prefracture values. No differences were seen between these 2 groups in lateral bending. NZ stiffness was also increased in both groups, without any intergroup differences. Instrumentation and HA restored sEZ approximately back to values similar to the intact state (Figure 6, Table 3).

#### Axial rotation

Instrumentation and HA approximately restored prefracture values for NZ and EZ and sEZ in axial rotation. No significant changes between the 2 groups were seen (Figure 6, Table 3).

## Discussion

Treatment of burst fractures has always been one of the most debatable subjects in traumatology. Therefore, there is a strong need for improved research in this field [32]. Fuzziness in fracture description and the absence of a universally used unique classification of these injuries may still play an important role in the persistence of many controversies, even in bone-healthy patients [5,33]. However, surgical treatment of incomplete burst fractures in patients with osteoporosis is even more challenging. Conventional surgical strategies with instrumentation for the osteoporotic spine is demanding because of the inherent risks of construct failure in poor bone stock [34].

Additionally, patients with osteoporosis often present multiple medical comorbidities and poorly endure open surgeries. Consequently, novel approaches and techniques have been developed to facilitate surgical treatment in these patients and reduce the incidence of construct failure [35].

The results of percutaneous vertebral body augmentation in osteoporotic compression fractures (OCF) without trauma has encouraged surgeons to extend the indications and use these techniques to restore anterior column even in traumatic fractures as stand-alone intervention or in combination with posterior instrumentation [9,10,14-16,20,21,34,36].

However, to our knowledge, only a few kinematic studies of vertebral augmentation [12] and the combination of augmentation and short pedicle instrumentation [7] have been performed so far.

Based on these reports, cement augmentation is considered to be able to restore the mechanical properties of the fractured vertebra, although the exact indications for clinical use remain unclear [12].

### Stand-alone vertebroplasty (VP)

In several studies, VP is described to be feasible and reliable for traumatic compression and even in burst fractures in osteoporotic spines [11,13,37]. Some authors even expand the indication and suggest VP alone for providing sufficient postlaminectomy stability [38].

VP and kyphoplasty are reported to be biomechanically equivalent methods for strengthening osteoporotic vertebrae [39]. Therefore, no further distinction between different augmentation techniques is made. Some effects of techniques other than VP, such as endplate reconstruction via balloon augmentation [35] could not be discussed based on our results.

In a previous biomechanical study, Lu et al. reported that injecting bioactive bone cement after burst fracture in a porcine model could restore mechanical properties [12].

This observation could only partly be supported by our results. We have seen a significant reduction of RoM (NZ + EZ) in flexion after VP in comparison to the fractured values. However, VP did not result in restoring prefracture values, whereas instrumentation resulted in more stability than the intact condition. After VP, NZ was increased by 16% to the prefractured values. Therefore, a persistence of traumatic instability may be expected from performing VP as a stand-alone procedure in real traumatic fractures. However, some authors concluded that most burst fractures without neurological involvement in bone-healthy patients can be safely and successful treated by conservative methods with comparable or even superior final outcome [1]. Therefore, the need for stability in these fractures in order to heal remains to be unknown.

Nevertheless, “burst fractures” do not equal “burst fractures”. There is no clear and common accepted definition of burst fractures and their instability. Comparing the published outcomes of the management in burst fractures may, however, be highly biased by comparing results of study cohorts of varying injury severities [33].

Evidence is provided that VP may support the anterior column especially in flexion, but does not restore pre-fracture stability. Given that burst fractures may successfully be treated conservatively, the need of isolated cement augmentation and the operative risk need to be discussed critically.

We therefore suggest distinguishing OCF (sintering) without relevant trauma from those with adequate trauma and osteoporosis. Distinction between the different entities of vertebral fractures in osteoporosis might result in better understanding of pathophysiology and will hopefully result in differentiated successful treatment strategies.

Based on the presented kinematic results, we would advocate against using VP as a stand-alone procedure in traumatic fractures in osteoporosis.

### Hybrid augmentation (HA)

Experiments by Oner et al. have shown that posterior instrumentation with distraction is able to reduce the displacement of the anterior and posterior but does not address a central impression of the fractured endplate. Central impression of the cranial endplate was significantly decreased by an additional kyphoplasty [14].

Posterior instrumentation in our study restored RoM of the intact 4 segmental specimens. However, without any anterior support, the instrumentation failed to restore prefractured values for stiffness in EZ in flexion.

Mermelstein et al. presented a significant alteration in pedicle screw-bending moments with flexion and extension with the injection of cement into the fractured vertebral body and short segment instrumentation. In conclusion, they indicated that the anterior column stability in burst fractures could be increased by HA [7].

The presented data support the lack of anterior column support in stand-alone bisegmental instrumentation even in incomplete burst fractures. The observed insufficiency of restoring stiffness in EZ may support the findings of Mermelstein et al. of an increase of screw-bending moments.

These kinematic findings may explain the well-known loss of correction with consecutive kyphosis in burst fractures after posterior instrumentation without anterior column support even in patients without osteoporosis [2].

However, additional cement augmentation of the fractured vertebra in our experimental setup was able to restore stiffness in elastic zone to prefractured values. This change in stiffness of EZ by additional augmentation in comparison to stand-alone posterior instrumentation again supports previous biomechanical findings on this technique [7].

Based on these kinematic findings, major primary stability seems to be achieved by the dorsal short segment pedicle construct. Additional augmentation may not result in further enhancement of primary stability but in support of the anterior column.

Clinical investigations describe a higher risk of instrument failure in short segment instrumentation with increased preoperative kyphotic deformity in osteoporotic patients [21]. VP is considered to increase spinal stability in patients with thoracolumbar burst fractures, to decreases the instrument failure rate, and to improve postoperative pain. This observation could indicate an insufficient anterior support.

Anterior column reconstruction by cement augmentation techniques in combination with short segment pedicle screw constructs is proposed to be a useful method for traumatic thoracolumbar spine fractures [14,35]. Therefore HA is appraised to provide immediate spinal stability and was suggested to be potentially equivalent to anterior reconstruction by some authors [16,21].

Our results partly support these clinical publications. Restoring mechanical stiffness properties in EZ in flexion may cause the described reduced failure rate of dorsal instrumentation after additional augmentation with HA.

However, we did not find any biomechanical indication that additive augmentation results in additional primary stability. Thus, some conclusions in literature about postoperative pain and outcome based on increase in primary stability could not be explained by our kinematic findings [16,21,40]. Furthermore, no prediction about long-term results of HA after load distribution can be made.

Nevertheless, the possibility of a minimally invasive reconstruction of the anterior column and even reposition of central impressions of the cranial endplate from posterior provides a promising potential for advanced treatment options in the future.

Particularly with regard to further development of bone cement which may alter mechanical properties in osteoporotic bone, improve mechanical resistance of resorbable cements or even provide osteoinduction, this technique may afford a less invasive treatment option for selected patients.

Consecutively, further work to demonstrate long-term stress and possible failure patterns are needed to reach the next step in understanding biomechanics of the presented hybrid treatment.

### Limitations

A limitation of this study, like all other cadaveric kinematic studies, is that the findings for initial stability are not able to predict in vivo results. No muscle trunk forces were applied and no preload was used which could alter the presented results. The study results, therefore do not allow any extrapolation to behavior under physiologic and repetitive loads.

Cement (PMMA) was inserted following the manufacturer’s advice. However, general conditions in cadaver vertebra are remarkably different from in vivo conditions. The influence of circulation, body temperature and other biological parameters in patients cannot be estimated.

Nevertheless, cement was inserted in a bipedicular manner and in some specimens, mild extrusion through the produced fracture was observed. Cement hardening was controlled; taking into account the different behavior of cement hardening at room temperature.

The used augmentation technique (vertebroplasty) and the experimental setup do not provide the possibility to correlate endplate restoration with kinematic values. This may be an important consideration as clinically described by other groups [14].

The used pedicle screws are known to cause some problems in osteoporotic vertebrae. However, we have chosen these to possibly reduce the impact of pedicle screws and focus on the effects of augmentation. The used setup only allows testing of primary stability. Further investigations for cyclic loading tested are needed.

## Conclusions

Stand-alone VP showed some support of the anterior column in our experimental setup. However, a persistence of traumatic instability was observed from performing VP in our trauma model. Therefore, we would advocate against using VP as a stand-alone procedure in traumatic fractures.

HA consisting of a combination of short segment instrumentation and VP did not change the primary stability already achieved by short segment instrumentation. Some additional support of anterior column and changes of kinematic values of EZ may indicate that additive augmentation possibly reduces the load of dorsal implants and possibly the risk of implant failure.

## Abbreviations

FSU: Functional spinal units; HA: Hybrid augmentation; VP: Vertebroplasty; RoM: Range of motion; NZ: Neutral zone; EZ: Elastic zone; BMD: Bone mineral density; PMMA: Polymethylmethacrylate; CT: Computer tomography; sNZ: Stiffness in neutral zone; sEZ: Stiffness in elastic zone; OCF: Osteoporotic compression fractures.

## Competing interests

Synthes GmbH, Switzerland provided the PMMA cement (Vertecem) and application kits used in this study. No further financial or material support was received. The Kuka Robot was supported by the Society for Arthroscopy and Joint Surgery (AGA). The authors declare that they have no competing interests.

## Authors’ contributions

RH conceived and planned the presented study. He observed and participated all presented biomechanical studies and drafted the manuscript. DG carried out the biomechanical studies, performed the statistical analysis and helped to draft the manuscript. MS observed and technically supported all biomechanical investigations. LM performed the radiological evaluation. MR participated in the design of the study and coordination. TV conceived of the study, and participated in its design and coordination and helped to draft the manuscript. All authors read and approved the final manuscript.

## Pre-publication history

The pre-publication history for this paper can be accessed here:

http://www.biomedcentral.com/1471-2474/14/360/prepub
